# Randomized controlled trial to assess the effectiveness of a videotape about radiotherapy

**DOI:** 10.1054/bjoc.2000.1536

**Published:** 2001-01-01

**Authors:** R Harrison, P Dey, N J Slevin, A Eardley, A Gibbs, R Cowan, J P Logue, V Leidecker, P Hopwood

**Affiliations:** Centre for Cancer Epidemiology, Kinnaird Road, Manchester, M20 9QL; The CRC Psychological Medicine Group, Christie Hospital NHS Trust; The Christie Hospital NHS Trust, Wilmslow Road, Manchester

**Keywords:** patient information, videotape, cancer, radiotherapy, anxiety, randomized controlled trial

## Abstract

In a randomized controlled trial, the additional provision of information on videotape was no more effective than written information alone in reducing pre-treatment worry about radiotherapy. Images of surviving cancer patients, however, may provide further reassurance to patients once therapy is completed. © 2001 Cancer Research Campaign http://www.bjcancer.com


British Journal of Cancer (2001) **84**(1), 8–10
© 2001 Cancer Research Campaign
doi: 10.1054/ bjoc.2000.1536, available online at http://www.idealibrary.com on IDEAL®

Short Communication

### Randomized controlled trial to assess the effectiveness of a videotape about radiotherapy

**R Harrison[1], P Dey[1], NJ Slevin[3], A Eardley[3], A Gibbs[1], R Cowan,[3] JP Logue,[3] V Leidecker[1] and P Hopwood[2]**

[1]Centre for Cancer Epidemiology, Kinnaird Road, Manchester, M20 9QL; [2]The CRC Psychological Medicine Group, Christie Hospital NHS Trust; [3]The Christie Hospital NHS Trust, Wilmslow Road, Manchester

**Summary** In a randomized controlled trial, the additional provision of information on videotape was no more effective than written information alone in reducing pre-treatment worry about radiotherapy. Images of surviving cancer patients, however, may provide further reassurance to patients once therapy is completed. © 2001 Cancer Research Campaign http://www.bjcancer.com

**Keywords:** patient information; videotape; cancer; radiotherapy; anxiety; randomized controlled trial

Cancer patients want detailed information on treatment (Meredith et al, 1996). In a randomized controlled trial (RCT), the booklet ‘Radiotherapy: a guide for patients and family’ given to patients booked for radiotherapy at the Christie Hospital reduced worry about some aspects of treatment (Eardley, 1988). We report a further RCT undertaken to determine whether additional benefits might follow the provision of a videotape based on the booklet.

Outpatients with head, neck, bladder or prostate cancer who required radical radiotherapy and had access to a videotape recorder were recruited between September 1997 and July 1998 following consultation with one of 4 clinical oncologists. Informed consent and baseline details were obtained by the investigator (RH) following which patients were randomly allocated to either an intervention group who received the videotape and written information (VT), or a control group who received written information only (WIO). The randomization schedule, administered by an off-site office, was stratified by sex, tumour site and source of referral.

All patients received the booklet which included information on planning and treatment and a question and answer section about common concerns, as well as a tumour-specific leaflet on side effects. Patients allocated to the intervention group also received the videotape which included: information on planning and treatment, interviews with treated patients about their experiences, and interviews with clinicians about side effects. All information materials were contained in a sealed opaque box.

Worry about radiotherapy was measured by a questionnaire completed by patients before randomization (baseline) and immediately before they started treatment (Eardley, 1988). Worry levels were categorized as follows: ‘very/quite worried’, ‘a bit/not really worried’ or ‘not at all worried’. Analysis was restricted to patients who responded at baseline and on the first day of treatment. Differences within groups were examined using the Wilcoxon Matched Pairs Signed Rank Test and between groups, using the Mann-Whitney test.

*Received 11 July 2000*
*Revised 13 September 2000*
*Accepted 18 September 2000*

*Correspondence to:* P Dey

General anxiety was measured using the anxiety subscale of the Hospital Anxiety and Depression Scale; this was completed by patients at baseline, on the first and last day of treatment, and 6 weeks after radiotherapy. For the purposes of this analysis, possible (summary score 8–10) and probable (summary score 11 or above) cases of anxiety have been combined (Zigmond and Snaith, 1983). Analysis was restricted to patients who responded at all 4 assessment points. General estimating equations were used to compare changes in anxiety over the study period.

In the previous trial, 24% of patients were very/quite worried about radiotherapy in general after receipt of the booklet (Eardley, 1988). It was estimated that 252 subjects were required to detect a reduction in the proportion of very/quite worried patients to 10% in the intervention group for a study power of 80% at the 5% two-sided significance level.

352 eligible patients were seen during the study period; 26 were not referred on to the investigator by consultants. Another 29 patients were excluded either because they had already viewed the videotape ($n$ = 25) or the randomization office could not be contacted ($n$ = 4). 23 (15.3%) patients refused to participate: 10 did not want more information, 9 could not wait and 4 did not offer a reason.

The remaining 274 patients were randomized; their mean age was 66 years and 240 (87.6%) were male. 141 patients (51.5%) were allocated VT and 133 (48.5%) WIO. The distribution of baseline characteristics across study groups is shown in Table 1. 6 patients allocated VT did not receive radiotherapy: 5 were found to have metastatic disease after recruitment. 4 patients failed to receive the correct information package but remained in their allocated group for analysis.

132 (93.6%) patients allocated VT and 132 (99.2%) allocated WIO responded to the question ‘How worried are you about radiotherapy in general?’ at baseline and on the first day of radiotherapy. 19 (14.4%) patients in the VT group and 20 (15.2%) in the WIO group were very/quite worried about radiotherapy at baseline. On the first day of radiotherapy, this proportion fell in both the VT group ($n$ = 16, 12.1%) and the WIO group ($n$ = 16, 12.1%). The reduction in worry levels from baseline was not significant in either the VT group ($Z = -0.63$, $P = 0.53$) or WIO group ($Z = -0.77$, $P = 0.44$); nor was the reduction in worry levels

**Table 1** Baseline characteristics of 274 patients recruited to the trial

| | Intervention (*n* = 141) | Control (*n* = 133) |
|---|---|---|
| Mean age in years (SD) | 66 (9.5) | 66 (9.1) |
| Range | 31–85 | 31–82 |
| Sex | | |
| Male | 122 (86.5%) | 118 (88.7%) |
| Female | 19 (13.5%) | 15 (11.3%) |
| Marital status[a] | | |
| Married/cohabiting | 114 (80.9%) | 95 (71.4%) |
| Other | 27 (19.1%) | 38 (28.6%) |
| Age left full time education (years)[b] | | |
| <17 | 120 (85.1%) | 116 (87.9%) |
| 17–19 | 13 (9.2%) | 10 (7.6%) |
| 20+ | 8 (5.7%) | 6 (4.5%) |
| Site of cancer | | |
| Head/neck | 41 (29.1%) | 39 (29.3%) |
| Bladder/prostate | 100 (70.9%) | 94 (70.7%) |
| Source of referral | | |
| Secondary | 65 (46.1%) | 61 (45.9%) |
| Tertiary | 76 (53.9%) | 72 (54.1%) |
| Very/quite worried about radiotherapy | 23 (16.3%) | 21 (15.8%) |
| Case of anxiety[b] | 40 (28.4%) | 36 (27.3%) |
| Median time in days from baseline to first radiotherapy session[c] | 21 | 21 |
| Range | 3–127 | 4–93 |

[a]not statistically significant and not associated with outcome.
[b]1 missing case in control group. [c]6 missing cases in intervention group.

significantly different across study groups (Mann Whitney U = 8675.0, $P = 0.94$) (Table 2).

The responses to questions about specific aspects of radiotherapy and proportionate changes in worry levels from baseline are shown in Table 2. Patients in the WIO group were significantly less worried about hair loss than those in the VT group (Mann Whitney U = 7365.5, $P = 0.02$).

118 (83.7%) patients allocated VT and 112 (85.5%) allocated WIO completed the anxiety subscale at all assessment points; at baseline, 29 (24.6%) patients in the VT group and 28 (25.0%) in the WIO group were anxious. General anxiety did not decrease in either study group until the last day of treatment but this fall was only maintained 6 weeks after treatment among those allocated the videotape: in the VT group, 30 (25.4%) subjects were anxious on the first day of radiotherapy, 19 (16.1%) on the last day and 18 (15.3%) 6 weeks after treatment; in the WIO group, 28 (25.0%) subjects were anxious on the first day of radiotherapy, 22 (19.6%) on the last day and 28 (25.0%) 6 weeks after treatment. The difference in the change in proportions of anxious subjects over all assessment points was not statistically significant ($\chi^2 = 3.32$, df = 3, $P = 0.34$).

In this cohort of cancer patients, the provision of information in either written or videotape format did not significantly reduce pre-treatment worry. However, the proportion of patients in this study who felt very or quite worried before treatment was lower than that previously reported among patients at the Christie (Eardley, 1988). This improvement may reflect the ongoing programme of communication skills training for consultants in this hospital. In addition, our study population consisted predominantly of elderly male patients who have fewer concerns and demand less information (Aass et al, 1996; Jones et al, 1999). It has been argued that older people are less conversant with videotape technology (Thomas et al, 1999) but a random sample of 14 patients in the VT group interviewed at the end of the trial had all viewed the videotape. A recent trial of videotape among a more heterogeneous cohort of patients undergoing radiotherapy or chemotherapy suggests that videotape reduces anxiety during treatment (Thomas et al, 2000). However, patients in this trial who were allocated videotape received more and different information than the control group and therefore the difference in outcome cannot solely be attributed to the medium. Other patient groups have not expressed a preference for audio-visual materials over written information (Coulter et al, 1999).

Patients receiving written information were significantly less worried about hair loss than those also receiving the videotape (Mann Whitney U = 7365.5, $P = 0.02$). Re-evaluation of the information materials revealed extensive coverage of this concern in

**Table 2** Comparison between study groups in change in worry from baseline to first day of radiotherapy

| Reason for worry | | | Change in the level of worry from baseline to first day of radiotherapy: decrease *n* (%) | no change *n* (%) | increase *n* (%) | Between group comparison statistical significance (Mann Whitney U) |
|---|---|---|---|---|---|---|
| 'Radiotherapy in general' | VT | (*n* = 132) | 22 (16.7) | 92 (69.7) | 18 (13.6) | U = 8675.0; *P* = 0.94 |
| | WIO | (*n* = 132) | 20 (15.1) | 95 (72.0) | 17 (12.9) | |
| 'In case treatment hurts' | VT | (*n* = 132) | 38 (28.8) | 75 (56.8) | 19 (14.4) | U = 8069.0; *P* = 0.23 |
| | WIO | (*n* = 132) | 24 (18.2) | 92 (69.7) | 16 (12.1) | |
| 'How long each treatment lasts' | VT | (*n* = 132) | 25 (19.0) | 82 (62.1) | 25 (19.0) | U = 8470.5; *P* = 0.66 |
| | WIO | (*n* = 132) | 30 (22.7) | 68 (51.5) | 34 (25.8) | |
| 'In case it's not safe for me' | VT | (*n* = 132) | 24 (18.2) | 87 (65.9) | 21 (15.9) | U = 8539.5; *P* = 0.74 |
| | WIO | (*n* = 132) | 24 (18.2) | 84 (63.6) | 24 (18.2) | |
| 'In case it's not safe for my visitors' | VT | (*n* = 132) | 18 (13.6) | 93 (70.5) | 21 (15.9) | U = 8454.5; *P* = 0.90 |
| | WIO | (*n* = 129) | 19 (14.7) | 89 (69.0) | 21 (16.3) | |
| 'About hair loss' | VT | (*n* = 130) | 17 (13.1) | 94 (72.3) | 19 (14.6) | U = 7365.5; *P* = 0.02 |
| | WIO | (*n* = 131) | 35 (26.7) | 80 (61.1) | 16 (12.2) | |
| 'About feeling sick' | VT | (*n* = 128) | 24 (18.8) | 83 (64.8) | 21 (16.4) | U = 8006.0; *P* = 0.52 |
| | WIO | (*n* = 130) | 17 (13.1) | 93 (71.5) | 20 (15.4) | |
| 'Feeling tense about treatment' | VT | (*n* = 128) | 29 (22.7) | 85 (66.4) | 14 (10.9) | U = 7416.0; *P* = 0.10 |
| | WIO | (*n* = 128) | 17 (13.3) | 95 (74.2) | 16 (12.5) | |
| 'Lying under machines' | VT | (*n* = 128) | 23 (18.0) | 89 (69.5) | 16 (12.5) | U = 7967.0; *P* = 0.54 |
| | WIO | (*n* = 129) | 18 (14.0) | 95 (73.6) | 16 (12.4) | |

the booklet but no direct reference to it in the videotape. As technology advances and it becomes easier to assemble and disseminate information, it is essential that as much attention is paid to quality as to presentation.

General anxiety, like worry about radiotherapy, did not decrease before commencement of treatment but did fall on the last day of treatment. This reduction was only maintained in the post-treatment period among those allocated VT. The absolute difference between study groups 6 weeks after radiotherapy of 9.7% was not significant ($\chi^2 = 3.41$, df = 1, $P = 0.065$) but this study was not designed to detect a significant difference of this magnitude for this endpoint. The concerns of cancer patients may change over time; following diagnosis, patients may be anxious about their forthcoming treatment but following treatment, they may be most anxious about their prognosis (Butow et al, 1997). The videotape contained footage of surviving cancer patients and we postulate that these images may provide longer-term reassurance to those more recently diagnosed. Future trials of videotape information should include such longer-term outcome measures.

### ACKNOWLEDGEMENTS

We thank Dr Sykes and all those who helped with the study at the Christie Hospital NHS Trust and Maelor General Hospital (North East Wales NHS Trust). RH was funded by the NHSE NW Research and Development Directorate. The video was funded by the Christie Hospital NHS Trust.

### REFERENCES

Aass N, Fossa SD, Dhal AA and Moe TJ (1997) Prevalence of anxiety and depression in cancer patients at the Norwegian Radium Hospital. *European Journal of Cancer* **10**: 1597–1604

Butow PN, MacLean M, Dunn SM, Tattersall MHN and Bayer MJ (1997) The dynamics of change: cancer patients' preference for information, involvement and support. *Annals of Oncology* **8**: 857–863

Coulter A, Entwistle V and Gilbert D (1999) Sharing decisions with patients: is the information good enough? *BMJ* **318**: 318–322

Eardley A (1988) Patients' worries about radiotherapy: evaluation of a preparatory booklet. *Psychol Health* **2**: 79–89

Jones R, Pearson J, McGregar S, Harpur Gilmour W, Atkinson JM et al (1999) Cross sectional survey of patients' satisfaction with information about cancer. *BMJ* **319**: 1247–1248

Meredith C, Symonds P, Webster L, Lamont D et al (1996) Information needs for cancer patients in West Scotland: cross sectional surveys of patients' views. *BMJ* **313**: 724–726

Thomas R, Deary A, Kaminski E, Stockton D and de Zueew N (1999) Patients' preference for videocassette recorded information: effect of age, sex and ethnic group. *European Journal of Cancer Care* **8**: 83–86

Thomas R, Daly M, Perryman B and Stockton D (2000) Forewarned is forearmed – benefits of preparatory information on video cassette for patients receiving chemotherapy or radiotherapy – a randomised controlled trial. *Eur J Cancer* **36**: 1536–1543

Zigmond A and Snaith R (1983) The Hospital Anxiety and Depression Scale. *Acta Psychiatry Scan* **67**: 361–370

*© 2001 Cancer Research Campaign*

